# Delving into public-expenditure elasticity: Evidence from a National Health Service acute-care hospital network

**DOI:** 10.1371/journal.pone.0291991

**Published:** 2024-03-04

**Authors:** Micaela Comendeiro-Maaløe, Manuel Ridao-Lopez, Enrique Bernal-Delgado, Andreu Sansó-Rosselló

**Affiliations:** 1 Data Science for Health Services and Policy Research, Instituto Aragonés de Ciencias de la Salud (IACS), Zaragoza, Spain; 2 Network for Research on Chronicity, Primary Care, and Health Promotion (RICAPPS), Instituto de Salud Carlos III, Madrid, Spain; 3 Department of Applied Economics, University of the Balearic Islands, Palma, Spain; 4 Aragon Health Research Institute (IISA), Zaragoza, Spain; 5 Models for Information Processing and Fuzzy Information (MOTIBO) Research Group, Balearic Islands Health Research Institute, Idisba, Mallorca, Spain; University of Bologna, ITALY

## Abstract

**Introduction:**

The sustainability of public hospital financing in Spain is a recurring issue, given its representativeness in annual public healthcare budgets which must adapt to the macroeconomic challenges that influence the evolution of spending. Knowing whether the responsiveness of hospital expenditure to its determinants (need, utilisation, and quasi-prices) varies according to the type of hospital could help better design strategies aimed at optimising performance.

**Methods:**

Using SARIMAX models, we dynamically assess unique nationwide monthly activity data over a 14-year period from 274 acute-care hospitals in the Spanish National Health Service network, clustering these providers according to the average severity of the episodes treated.

**Results:**

All groups showed seasonal patterns and increasing trends in the evolution of expenditure. The fourth quartile of hospitals, treating the most severe episodes and accounting for more than 50% of expenditure, is the most sensitive to quasi-price factors, particularly the number of beds per hospital. Meanwhile, the first quartile of hospitals, which treat the least severe episodes and account for 10% of expenditure, is most sensitive to quantity factors, for which expenditure showed an elasticity above one, while factors of production were not affected.

**Conclusions:**

Belonging to one or another cluster of hospitals means that the determinants of expenditure have a different impact and intensity. The system should focus on these differences in order to optimally modulate expenditure not only according to the needs of the population, but also according to the macroeconomic situation, while leaving hospitals room for manoeuvre in case of unforeseen events. The findings suggest strengthening a network of smaller hospitals (Group 1)–closer to their reference population, focused on managing and responding to chronicity and stabilising acute events–prior to transfer to tertiary hospitals (Group 4)–larger but appropriately sized, specialising in solving acute and complex health problems–when needed.

## Introduction

In terms of Spanish government expenditure, healthcare is the most important welfare system directly managed by regions, accounting for between 3.9% and 9.5% of the regional GDP. Within this total, public hospital expenditure (PHE) is by far the largest share, accounting for 62.4% of total public healthcare expenditure in 2015 [1]. The sustainability of public hospital financing in Spain is therefore a recurring issue, given its representativeness in annual public healthcare budgets which have to adapt to the ever-changing macroeconomic challenges that strongly influence the evolution of expenditure [2]. The Spanish National Health System (SNHS) is conceptually characterised as a national health service that is essentially financed by taxes, although there are cost-sharing arrangements for some services, notably outpatient prescription drugs. Since the approval of the General Health Act in 1986 [3] the SNHS has consisted of 17 regional healthcare systems, each of which is a single-payer system covering a broad basket of services. In each of these regions, the population is allocated to administrative healthcare areas and registered to primary care physicians who have a gatekeeping role, except in the case of urgent care. Hospital care is mainly provided by public hospitals, which are reimbursed for all their real costs incurred in the previous year through global lump-sum budgets [4] and cover all types of demand coming from their reference population. Since 2001, when the process of devolution of healthcare to the Autonomous Communities was fully completed [5], total public expenditure on healthcare has increased at a much higher rate than GDP growth (85.1% vs. 54.4%); public hospital expenditure has increased at an even faster rate (120%). Although some of the existing literature has examined the determinants of public healthcare expenditure, all the data used is prior to the fully completion of the healthcare devolution process, and the units of analysis are regions as opposed to hospitals [6–9].

Acute hospital provision in the SNHS is graded from ‘tertiary care hospitals’, which imply the availability of a wide range of highly specialised care and technically sophisticated services, to ‘general hospitals’, which provide inpatient care in each of the healthcare areas. The minimum basket of services in general hospitals includes internal medicine, general surgery, and minor surgical specialities. Depending on the severity of the condition, general hospitals may refer patients to a tertiary hospital.

In this sense, there is an international classification [10, 11] that divides hospitals into three types according to their degree of specialisation and complexity of the care they provide. In Spain, the Ministry of Health has its own classification, which takes into account five types of hospitals according to a combination of characteristics, such as number of beds, provision of technological equipment, human resources and the severity of the episodes attended to [12]. Beyond this classification, since the process of devolution of healthcare to the regions was fully completed, it can be noted that some regions have their own classification of hospitals, drawing a heterogeneous scenario that prevents comparisons when dealing with specific studies on hospital performance. Therefore, it seems necessary to establish an ad hoc clustering of hospitals according to a specific characteristic, depending on the area of interest of the performance assessment.

Although there are many studies in which it is common to compare hospitals on the basis of performance indicators per se [13–17], and also studies that compare indicators based on the classic distinction between teaching and non-teaching hospitals [18, 19] or between public and private hospitals [20–23], it is more difficult to find studies in which the comparison of performance indicators is based on a closer to optimal classification of hospitals, for example when technical efficiency and quality need to be assessed together [24]. In this sense, building on two previous studies that assess public hospital expenditure cross-sectionally and dynamically [2, 25], this paper aims to assess whether the responsiveness of hospital expenditure to its determinants (need, utilisation, and quasi-prices or costs of factors of production) varies according to the type of hospital. More specifically, we use a dynamic approach that evaluates public hospital expenditure per group of hospitals, taking into account an ad hoc classification that clusters them according to the average severity of the episodes attended to, i.e. “severity” as a factor that is closely related to the “complexity” of the episode and consequent services provided in terms of the intensity of use of highly developed technological equipment, availability of a wide range of medical and surgical specialities, and qualified personnel [26].

Given that the grouping of hospitals has been based on a distinctly characterised episode profile in terms of its complexity, the main objective is to find out whether this motivates the determinants explaining hospital spending to affect differently from previous findings [2, 25]. Based on the management of hospital resources according to the specific profile of the demand to be attended to, the underlying assumption is that the determinants assessed should have the same impact on PHE irrespective of whether the hospital belongs to one or another group of hospitals.

## Methods and design

### Design and population

An observational study based on clinical and administrative microdata, representing public hospital expenditure in all acute-care hospitals in the SNHS from January 2003 to December 2015. A total of 59 million anonymised episodes were analysed over the period. The information of interest (i.e. dependent variable and regressors) from each individual episode was aggregated by hospital at a monthly level.

### Sources of information

Three sources were linked for this study: 1) the microdata infrastructure of anonymised clinical and administrative hospital data maintained by the AtlasVPM project [27], which contains information on the date of admission, age and sex of the patient, burden of disease, hospital admissions, case-day surgeries and length of stay; 2) the APR-DRGs grouper (v32 licensed by ©3M) which provides information on the severity of each episode as a proxy; and, 3) the Official Statistics of Health Establishments providing Inpatient Care (ESCRI) [28] which provides information on the workforce, equipment, and spending.

### Dependent variable

The dependent variable was defined as the monthly inter-annual growth rate of public hospital expenditure adjusted for patient severity. PHE was built from the unitary costs per admission per month, adjusted by APR-DRG severity weights. In the absence of hospital analytical accounts, our dependent variable–or equivalent expenditure for each episode and hospital–was estimated by dividing the official expenditure figure [28] by the sum of the APR-DRG weights (i.e. for the total sum of episodes) and then multiplied by the specific weight of each episode. In addition, in order to work with a homogeneous monthly time series, PHE was deflated using 2003 as the base year.

### Hospital clustering

SNHS acute-care hospitals were stratified into four groups: the variable containing the hospital APR-DRG weights, by proxying the average severity of the episodes attended to at each hospital, was divided into quartiles. Group 1, hospitals attending to the least complex episodes (n = 66), with an average APR-DRG weight less than 0.753; Group 2, corresponding to hospitals attending to the middle-low complex cases (n = 69), with average APR-DRG weight ranging from 0.754 to 0.807; Group 3, gathering hospitals attending to middle-high complex episodes (n = 69), with average APR-DRG weight ranging from 0.808 to 0.874; and finally Group 4, hospitals attending the most complex episodes (n = 69), namely hospitals with an average APR-DRG weight above 0.875.

### Regressors: The factors explaining public hospital expenditure

Based on the analysis of the classic micro-economic theory [29] and the preceding studies [2, 25] the main factors affecting PHE were collected in three groups: attended population needs, utilisation (the quantities), and quasi-prices or costs of factors of production (from the producers’ perspective, price equals direct and indirect costs of factors of production when the margin of benefit is zero, which is the case for the reimbursement model of public hospitals in the SNHS):

i) *Need* gathered the systems response to the hypothetically ‘real need of patients’ and was defined by using four different variables characterising the population attended to: a) percentage of men; b) percentage of people aged 65 and over (retirement age); c) percentage of elderly (people aged 75 and over); and, d) morbidity, as the cumulative number of hospitalisations for hip fracture, acute myocardial infarction, ischemic stroke, and surgically treated cancers of the colon, lung, or breast, in patients aged 40 and over. These hospitalisations reflect differences in ‘real need’ as they are not influenced by supply-side factors [25, 30].
ii) *Utilisation* was defined using three variables collecting the different types of hospitalisations produced in the period of study (i.e. quantities), distinguishing between medical hospitalisations, surgical admissions, and outpatient case-day surgeries (CDS).
iii) *Quasi-prices* were approximated according to two sub-sets of factors: length of stay and structural features; a) average length of stay (ALOS), distinguishing between ALOS in medical and surgical admissions; and b) structural features reflecting number of hospitals in each group, beds, and the evolution of the supply of professionals (i.e. medical staff and nursing staff), and the system’s teaching capacity (i.e. medical residents–MIR). In this work, although ALOS could be expected to proxy the cost of an episode [2, 25, 31, 32], as episode severity was previously adjusted (see endogenous variable), it only informed about ancillary and lodging costs.

### Analyses

SARIMAX models (i.e. Seasonal Autoregressive Integrated Moving Average with eXogenous factors) were used to estimate the elasticities of need-adjusted PHE to the utilisation and cost factors in each of the four clusters of hospitals. SARIMAX models are used to capture both the linear relationships between a dependant variable and a set of conditioning variables (regressors), as well as the dynamics of the response (autoregressive and moving average components). Moreover, both the moderate time span and number of regressors suggest that other techniques, such as VAR models, should be ruled out because they imply a high number of parameters and very few degrees of freedom in our setting. The SARIMAX models were specified with 12 explanatory variables and estimated with monthly time series from January 2003 to December 2015 (156 observations; 144 after taking seasonal differences), aimed at assessing the response of need-adjusted PHE to the evolution of the underlying factors (i.e. changes in its elasticity to quantities and costs and consistency over time) in each of the groups of hospitals.

Given the multiplicative relationship of the regressors, as expenditure is a multiplicative function of quantities of goods or services (Q) and their costs (P), time-series of PHE were transformed into logarithms, so that the multiplicative relationship becomes linear. Hence, logarithms were also applied to the regressors, except for the variables obtained from ratios (i.e. need variables collecting the percentages). Moreover, to ensure stationarity, the dependent variable was seasonally differenced, as were the (log-transformed) regressors. Thus, the resulting dependent variable should be interpreted as an inter-annual growth rate.

Formulation of the final specified models is as follows:

$$\Delta_{12}Y_{t}=\mu_{0}+\sum_{s=1}^{12}\lambda_{s}D_{st}+\sum_{n=1}^{N}\beta_{n}\Delta_{12}X_{nt}+\sum_{k=1}^{K}\gamma_{k}\Delta_{12}Q_{kt}+\sum_{h=1}^{H}\alpha_{h}\Delta_{12}F_{ht}+\sum_{j=1}^{J}\omega_{j}DU_{jt}(T_{i})+\sum_{i=1}^{I}\delta_{i}D_{it}(T_{i})+\frac{\theta (L)}{\phi (L)}\varepsilon_{t}$$

Where: Δ_12_ is the seasonal difference operator, so that Δ_12_ *Y*_*t*_ = *Y*_t_ − *Y*_*t*-12_

*Y*_*t*_: the endogenous variable, is the logarithm of the severity-adjusted public hospital expenditure in period t, deflated at 2003 (base year)

*D*_*st*_: are monthly seasonal dummies. They would be equal 1 if t belongs to month-s and zero otherwise.

*X*_*nt*_: N regressors which characterize the population of each attended episode at period t (need factors)

*Q*_*kt*_: K regressors capturing the use of healthcare hospital services at period t (logarithm of utilisation factors)

*F*_*ht*_: H regressors capturing factors of production which approach to prices of hospital healthcare services provided at period t (supply factors or prices)

*DU*_*jt*_(*T*_*j*_): J dummy variables capturing structural changes. They would be equal 1 if t ≥ T_j_ and zero otherwise.

*D*_*it*_(*T*_*i*_): I dummy variables for the treatment of outliers. They would be equal 1 if t = T_i_ and zero otherwise.

$\varepsilon_{t}\;iidN\left(0,\sigma_{\varepsilon }^{2}\right)$ is a white noise process.

*θ*(*L*) = 1 − *θ*_1_ *L* −⋯ −*θ*_*q*_ *L*^*q*^ and *ϕ*(*L*) = 1 − *ϕ*_1_ *L* −⋯ −*ϕ*_*p*_ *L*^*p*^ are the Moving Average and Autoregressive polynomials, respectively, and L is the lag operator such that, for any variable *x*_*t*_, *L*^*j*^ *x*_*t*_ *= x*_*t−j*_

*μ*_0_, *λ*_*s*_, *β*_*n*_, *γ*_*k*_, *α*_*h*_, *ω*_*j*_, *δ*_*j*_, *θ*_*r*_ and *ϕ*_*l*_ are unknown parameters to be estimated.

The optimal final models for each cluster of hospitals were chosen according to the Akaike and Hannan-Quinn information criteria [33] and validated through the Lagrange Multiplier (LM) autocorrelation test and the Bera-Jarque normality test of residuals [34]. Specifications were coded and estimated using the cross-platform software package for econometric analysis, gretl (Gnu Regression Econometrics and Time Series Library) v.1.9.4. The estimation procedure was exact maximum likelihood.

### Ethical issues/statement

This study, observational in design, using retrospective anonymised, non-identifiable and non-traceable data, was conducted in accordance with the amended Helsinki Declaration, the International Guidelines for Ethical Review of Epidemiological Studies, and Spanish laws on data protection and patients’ rights. This study implies the use of pseudonymised data, using double dissociation (i.e. in the original data source and once data were stored in the database for analysis), thereby effectively impeding patient re-identification.

## Results

**Fig 1:**
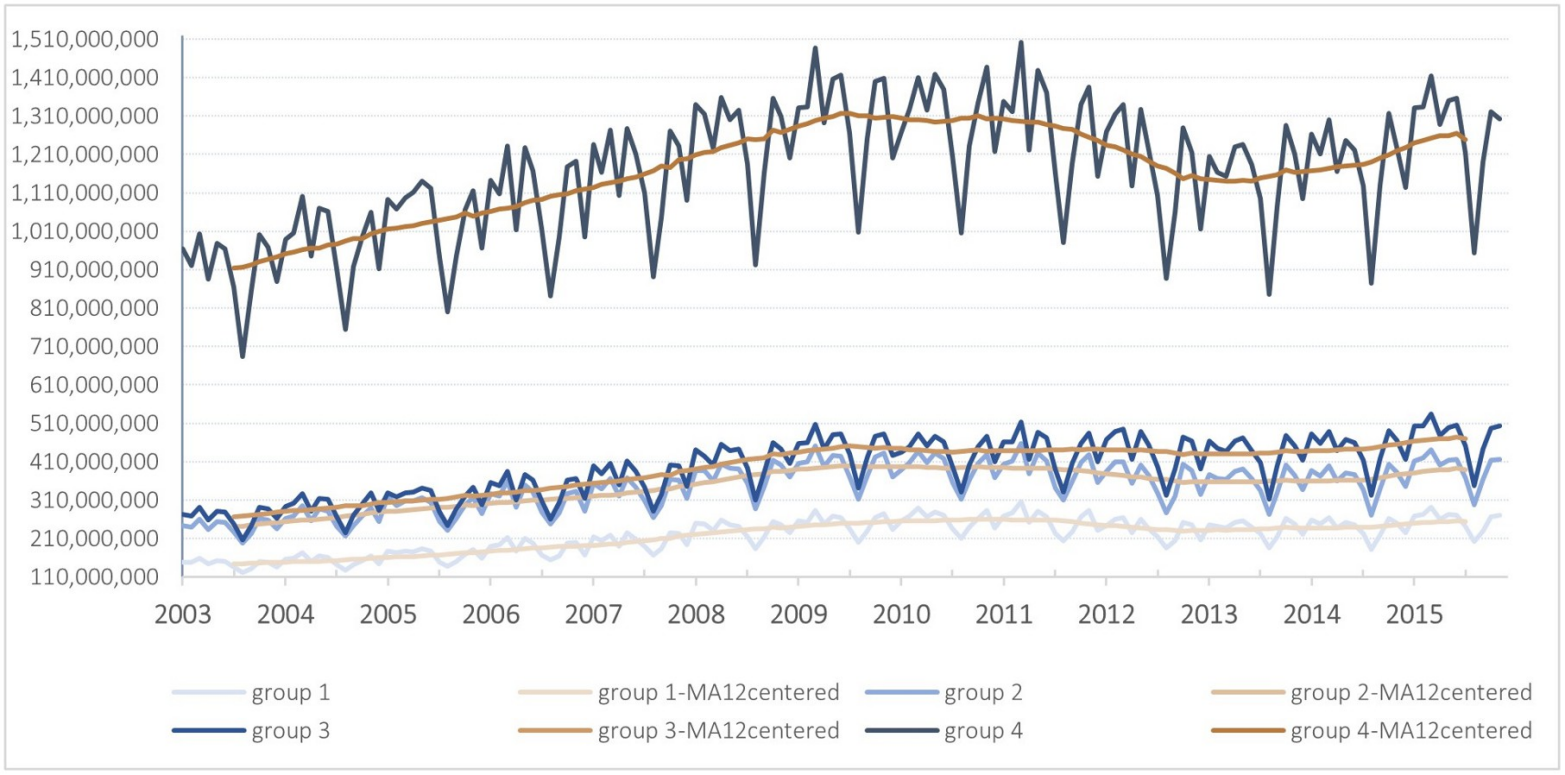
Evolution of monthly deflated need-adjusted public hospital expenditure by group of hospitals. Hospitals are clustered according to the average complexity of the episodes they attended to. Group 1 includes hospitals that treat the least complex cases on average, up to Group 4, which includes hospitals that treat the most complex episodes of care. The overlapping line “MA12centred” stands for the 12^th^-order centred moving average of hospital expenditure.

In the SNHS, the classification of hospitals according to the severity of the patients they treat enables us to observe differences in the volume of their expenditure and the pattern of its interannual variation over the years of the study. Group 1 (the group dealing with the least severe episodes), which accounted for 9.2% of the total sum of PHE at the beginning of our study period (2003), increased by 76.6% over the period (€110,070 million in constant terms) and accounted for 10.8% of the total sum of PHE in 2015. In contrast, Group 4 (those attending to the most severe and complex episodes) accounted for 58.4% of the total sum of PHE in 2003, increased by 36.5% over the period (€333,609 million in constant terms) and accounted for 52.9% of the total sum of PHE in 2015. Groups 2 and 3 accounted for 15.4% and 17%, respectively, of the total amount of PHE in 2003. Group 2 increased by 61.6% (€148,287 million in constant terms) to account for the 16.5% of the total sum of PHE in 2015, while Group 3 increased by 76.6% (€203,695 million in constant terms) to account for 19.9% of the total sum of PHE in 2015 (Fig 1).

### Evolution of underlying factors of PHE: Descriptive statistics

On a monthly average, the hospitals in Group 4, which have the highest volume and most severe episodes of care, recorded 91,121.2 non-surgical admissions, 56,683.2 surgical hospitalisations, and 35,759 outpatient case-day surgeries, with an associated cost of €1,160.5 million in constant 2003 terms. In contrast, the hospitals in Group 1, which have the lowest volume and least severe episodes of care, recorded 25,295.6 non-surgical admissions, 12,170.3 surgical hospitalisations, and 11,954.2 outpatient case-day surgeries, with an associated cost of €216.4 million in constant 2003 terms. Intermediate hospital Groups 2 and 3 recorded, respectively, a monthly average of 37,105.2 and 38,474.2 non-surgical admissions, 18,987.4 and 20,340.5 surgical hospitalisations, and 18,594 and 16,144.3 outpatient case-day surgeries, accounting for €343.9 and €392.3 million in constant 2003 terms (see S1 Table 1 in S1 Appendix).

Notwithstanding the noticeable seasonal pattern and increasing trend in the evolution of PHE, its determinants evolved differently according to the group of hospitals during the period. Observing the evolution of the factors underlying PHE in each of the groups is crucial to later understanding their marginal effects.

Regarding the evolution of the ‘need’ factors (see S1 Fig 1 in S1 Appendix—Evolution of monthly *need factors* by group of hospitals, Jan2003-Dec2015), the percentage of men showed a similar pattern, fluctuating between 45% and 47% over the period in Group 1, but increasing in the other groups from 2009 onwards. The percentage of people over 65 years of age showed a steady increase in all groups, with a higher proportion of older people treated in Group 3 and the lowest in Group 4. The proportion of the eldest people followed the same pattern in the Groups of hospitals 1 to 3, showing an increasing trend until 2009, but stabilising thereafter. However, Group 4 had the lowest proportion of eldest people. Finally, the evolution of patients’ comorbidities showed a slight growth, especially in Group 4.

**Fig 2:**
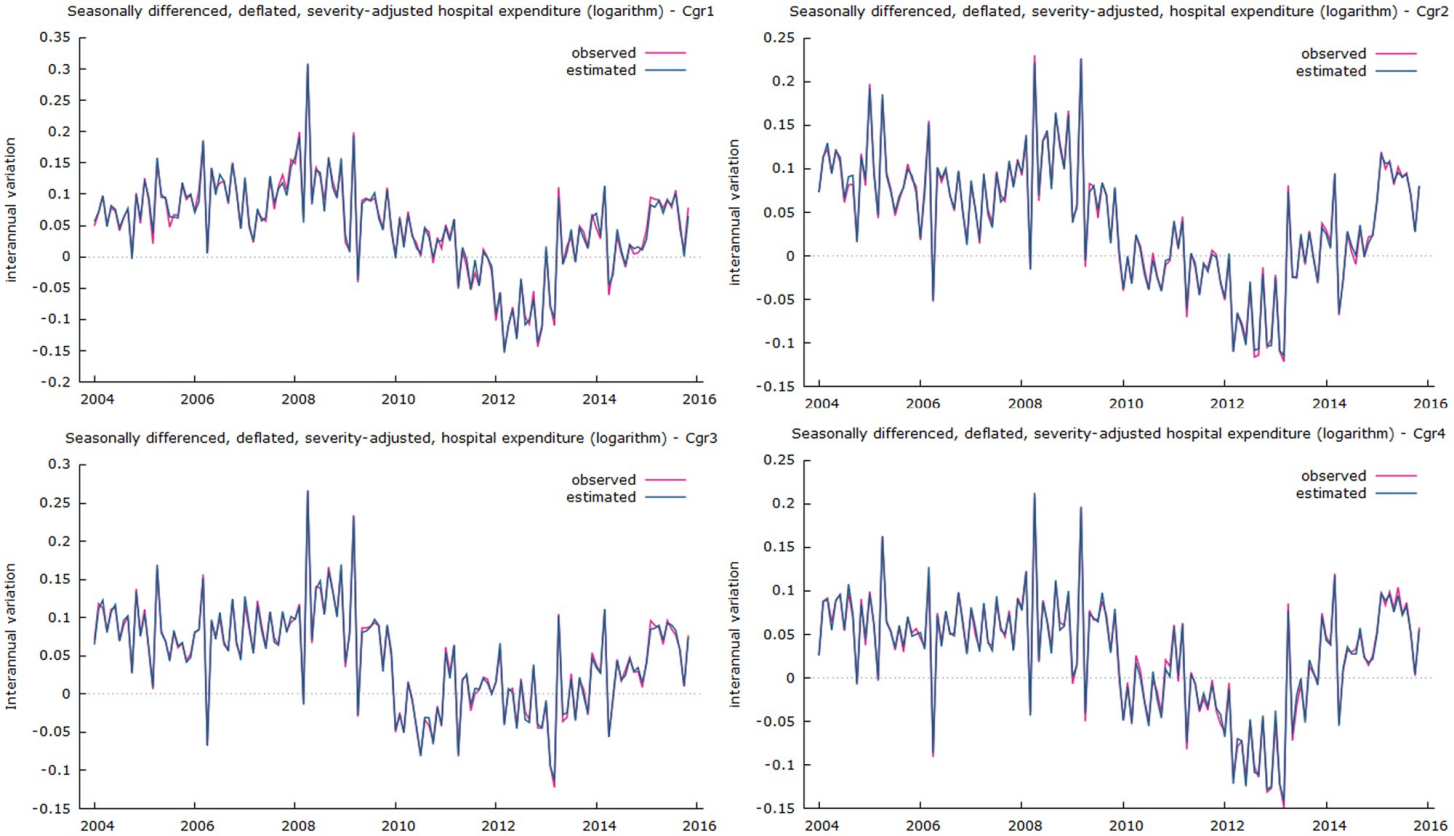
Observed to predicted need-adjusted public expenditure interannual growth in hospital care. Goodness of fit of models: Cgr1- corrected R^2^ = 0.9886, model Cgr2- corrected R^2^ = 0.9928, model Cgr3—corrected R^2^ = 0.9930, model Cgr4—corrected R^2^ = 0.9925).

Regarding the evolution of the utilisation factors proxying ‘quantities’, overall interannual monthly surgical and non-surgical hospital activity increased by 32% in Group 1, 31% in Group 2, 28.4% in Group 3 and 14.3% in Group 4 (see S1 Fig 2 in S1 Appendix—Evolution of monthly *utilisation factors* by group of hospitals, Jan2003-Dec2015). In relative terms, these figures were mainly driven by a large increase in case-day surgeries, particularly in Groups 2 and 1, with rises of 337% and 303%, respectively. Case-day surgeries also doubled in Group 3 and increased by 120% in Group 4. In contrast, surgical hospitalisations increased mainly in the hospital Groups 3 and 4, by 14.7% and 9%, respectively, but only 5.5% in Group 1, and 3.3% in Group 2. Non-surgical admissions evolved differently. Groups 1 to 3 initially showed an increasing trend until 2009, then dipped coinciding with the financial crisis, subsequently recovering from 2013 onwards, with an overall increase of 14.5%, 9% and 14.8%, respectively. On the other hand, hospitals in Group 4, after showing an increasing trend until 2008, started a steady decreasing trend with an overall decrease of 3.2%.

Finally, in the case of factors of production proxying ‘quasi-prices’ (see S1 Fig 3a in S1 Appendix—Evolution of factors of production (*quasi-price factors*) by group of hospitals, Jan2003-Dec2015; and S1 Fig 3b in S1 Appendix—Evolution of factors of production (human resources) by group of hospitals in relation to beds, Jan2003-Dec2015;), the time trends in functioning beds remained stable in Groups 1 and 2, although they showed two pronounced structural changes in 2006 and 2012. In total, the number of functioning beds in these two hospital groups increased by 1.3% and 4.1%, respectively. Groups 3 and 4, however, showed a continuous decrease (7.8% and 14.7% respectively). The number of fully contracted medical staff showed a marked upward trend in Groups 1 to 3, although this slowed down from 2012 onwards. In total, these groups increased by 37.6%, 42.3% and 24.2%, respectively. Group 4 also showed a marked upward trend, but with a turning point in 2010, followed by a dip and a new recovery in its figures from the beginning of 2015. In summary, the ratio of medical staff to functioning beds increased over the period in all hospital groups. However, in Groups 3 and 4, it remained stable from 2012 onwards.

In the case of nursing professionals, all four groups of hospitals reduced recruitment from 2010 onwards. This interrupted the upward trends observed at the beginning of the period, particularly in Group 4, where the reduction brought the number of professionals to a figure 3% lower than at the beginning of the period. In general, however, the ratio of nursing staff to beds in hospital Groups 1 to 4 increased by 16.5%, 18.6%, 14.2% and 14.1%, respectively. The number of MIR students increased steadily over the period in all hospital groups, but more markedly in Groups 1, 2 and 3 (126.8%, 111.6% and 73.1%, respectively). In the group of hospitals treating the most severe episodes, these students increased by 14.2%. In summary, the teaching capacity (ratio of MIR to medical professionals) also increased significantly in Groups 1 to 3 (64.8%, 48.7% and 38.5%, respectively), but only increased slightly, 4.7%, in Group 4. Finally, both types of ALOS (medical and surgical) showed decreasing trends in all hospital groups, but particularly in Group 4.

### Dynamic analysis of the factors underlying PHE: SARIMAX modelling

From an aggregate perspective and after controlling for need variables, utilisation factors were observed as the common denominator of all groups explaining PHE growth over the whole period (T = 144 months), with surgical hospitalisations showing the highest magnitude of association. The exception is the group of hospitals dealing with the least severe episodes, where medical hospitalisations showed the most relevant association. S1 Table 2 in S1 Appendix shows the estimated coefficients of the models. A summary of the main results is shown in Table 1, where some of the variables and coefficients are aggregated (additive re-parameterisation). For instance, the table shows the sum of the coefficients of the utilisation factors (surgical hospitalisations, medical hospitalisations, and outpatient case-day surgery); this value, 1.06, should be interpreted as the overall effect of an increase of 1% in these factors.

**Table 1:** Deflated, severity adjusted, public hospital expenditure by subgroup of hospitals according to the severity of the episodes attended to (re-parametrised coef.).

SARIMAX regressions	Cgr1		Cgr2		Cgr3		Cgr4	
	SARIMAX(0,0,3)(0,1,1)_12_		SARIMAX(0,0,7)(1,1,0)_12_		SARIMAX (1,1,0)_12_		SARIMAX (1,1,0)_12_	
	Coefficient	Std. Dev	Coefficient	Std. Dev	Coefficient	Std. Dev	Coefficient	Std. Dev
Intercept	**0.0408**	0.0055	**0.0319**	0.0039	**0.0671**	0.0064	**0.0529**	0.0045
		(0.0000)		(0.0000)		(0.0000)		(0.0000)
Percentage of men	0.1693	0.1841	-0.2660	0.1996	**0.3107**	0.1783	-0.4209	0.2697
		(0.3577)		(0.1827)		(0.0814)		(0.1186)
Percentage over 65	**1.3205**	0.2254	**-0.3945**	0.2377	0.0058	0.2272	0.2658	0.2685
		(0.0000)		(0.0970)		(0.9796)		(0.3222)
Percentage over 75	**-1.3340**	0.2276	**0.5157**	0.2787	**0.4628**	0.2089	0.3234	0.2885
		(0.0000)		(0.0643)		(0.0267)		(0.2623)
Ln (morbidity)	**-0.0318**	0.0174	0.0050	0.0179	0.0102	0.0172	-0.0572	0.0252
		(0.0668)		(0.7803)		(0.5545)		(0.0235)
Utilisation factors (after re-parameterisation)	**1.0632**	0.0260	**1.0128**	0.0216	**0.9851**	0.0206	**1.0988**	0.0266
		(0.0000)		(0.0000)		(0.0000)		(0.0000)
Length of stay (after re-parameterisation)	0.0844	0.5051	0.0538	0.0361	0.0241	0.0354	**0.1421**	0.0381
		(0.0948)		(0.1182)		(0.9929)		(0.0002)
Beds per hospital (after re-parameterisation)	**-0.2739**	-0.0372	0.0662	0.3006	**0.1608**	0.0352	**1.0809**	0.0410
		(0.0066)		(0.6388)		(0.0325)		(0.0000)
Professionals in context to beds (after reparameterisation)	0.0212	0.0523	0.0922	0.0479	**0.1579**	0.0511	**0.3729**	0.1403
		(0.6853)		(0.0541)		(0.0020)		(0.0079)
Std. Dev. dependent variable	0.0737		0.0701		0.0639		0.0652	
Std. Dev. Model	0.0072		0.0053		0.0049		0.0052	
R squared	0.9905		0.9943		0.9942		0.9937	
R squared corrected	0.9886		0.9928		0.9930		0.9925	
Akaike info criterion	-949.19		-1023.59		-1059.99		-1045.83	
Hannan-Quinn	-916.68		-985.06		-1028.68		-1014.52	
Autocorrelation test	11.8873	(0.2927)	9.94486	(0.2689)	14.6662	(0.1983)	11.6591	(0.3898)
Normal distribution of residuals test	1.8660	(0.3934)	0.5218	(0.7704)	3.8882	(0.1431)	0.7671	(0.6814)

(Note: Bold coefficients stand for statistical significance. Figures in parenthesis stand for p-values).

Overall, an additive re-parameterisation would translate into a 1% increase in the inter-annual growth rate of hospital activity (utilisation) into a 1.1% increase in the inter-annual growth rate of PHE in Groups 1 and 4, showing a statistically significant elasticity above 1 (performed beta coefficients sum test p-value < 0.0001) (Table 1). Meanwhile, the growth rate of PHE in Groups 2 and 3 showed a unitary elasticity with respect to hospital activity.

The number of functioning beds per hospital in each group turned out to be a determinant factor explaining the evolution of the inter-annual growth rate of PHE among the factors of production (cost factors or quasi-prices), especially in Group 4, where it showed a statistically significant elasticity above one. On average, a 1% increase in the inter-annual growth rate of functionating beds per hospital would lead to a 1.1% increase in the inter-annual growth rate of PHE.

The ALOS for all types of admissions was only significant in the group of hospitals dealing with the most complex episodes of care (Group 4). A 1% increase in its interannual growth rate would translate into a 0.14% increase in the interannual growth rate of PHE.

Finally, the variables capturing human resources in relation to functioning beds were only statistically significant in hospitals dealing with episodes above the median of the severity distribution (Groups 3 and 4).

## Discussion

This dynamic assessment shows how utilisation factors (inpatient activity–medical and surgical–and outpatient case-day surgery) were the main drivers explaining the interannual growth rate of PHE in the SNHS, irrespective of the profile of patients treated in the four different hospital clusters; although utilisation (the quantities) showed the strongest effects in Groups 1 and 4 –those dealing with the least and the most severe episodes of care. However, in the fourth group, the variation of the factors of production or quasi-prices also showed some significant effect on the interannual growth rate of PHE, especially the number of functioning beds per hospital, with a unitary elasticity close to the elasticity shown by the quantities.

### Utilisation vs. quasi-prices when clustering hospitals by severity of episodes: Explanation of results

The findings of this work underline the results obtained in previous research carried out at the SNHS level [2, 25], where the hospital activity itself, i.e. the quantities or so-called utilisation factors (inpatient medical activity, surgical activity and outpatient case-day surgery) showed a greater influence in explaining PHE than variables related to the cost of factors of production or quasi-prices, but only in hospitals below the 75^th^ percentile of the distribution of complexity attended to, i.e. those belonging to Groups 1, 2 and 3. In fact, delving into the specificities of clustering by episode severity, led to the emergence of nuances enabling a better understanding of the influence of the determining factors, especially improving the interpretation of the influence of the factors of production on PHE.

When assessing the extreme clusters, i.e. the groups dealing with the least and most severe episodes of care, it can be observed that while utilisation factors as a whole showed a similarly stronger effect in explaining PHE (both with an elasticity above 1) compared to the mid distribution groups of hospitals, the cost of factors of production showed significant differences between them. Group 1 found its most important determinant of PHE in medical admissions, whereas the factors capturing the use of factors of production were not statistically significant. This type of acute care hospitals, in addition to the treatment of minor surgeries and uncomplicated acute episodes, are mainly focused on the treatment of decompensations of chronic health conditions or fragile patients, mostly with an older average age. On the other hand, PHE in Group 4 showed surgical hospitalisations to be the strongest determinant among the utilisation factors, but was also shown to be unitary elastic to the number of functioning beds and to obtain–compared to the other hospital groups–the strongest effect in terms of changes in the average length of stay of episodes and decisions concerning human resources.

Meanwhile, hospitals above the mid distribution of complexity, Groups 3 and 4, showed more similarities in terms of which determinants within the quasi-prices positively influenced PHE, albeit with different intensities, whereas in Groups 1 and 2 these factors showed no statistical influence at all. The effect of the number of functioning beds per hospital, which explained the interannual growth rate of PHE in Groups 3 and 4, was not present in Groups 1 and 2. Length of stay was only significant in Group 4, and the effect of human resources was twice as large in Group 4 as in Group 3. While a unitary percentual variation in the interannual growth rate of the ratios of staff to functioning beds would imply a variation of 0.37% in the interannual growth rate of PHE in Group 4, it would imply a variation of 0.16% growth in Group 3. Teaching capacity also showed a greater effect in Group 4 than in Group 1.

### Consistency with current knowledge

The importance of utilisation [35–38], quasi-prices [39–41] or the combination of both types of determinants [42] in the evolution of PHE is well documented. Moreover, the influence of the utilisation factors has been the outcome of previous articles [2, 25]. However, there are no studies measuring the effect of the determinants of PHE in an NHS setting such as ours, with a population administratively allocated to hospitals and without any rivalry for patients between providers, assessed by clusters of hospitals and over such a long period of time. In other contexts, such as Medicare, although not concerned with assessing the determinants of expenditure as we are, some studies have been found that assessed differences in costs by hospital groups, although essentially comparing teaching and non-teaching hospitals. These studies found slightly higher costs per patient over 30 days of hospitalisation in teaching hospitals [18]; higher marginal costs for general, vascular, or orthopaedic surgery in teaching hospitals [43], but similar costs in both types of hospitals for abdominal aortic aneurysm repair or lung resection [44], or even lower costs for acute exacerbations of COPD in teaching hospitals [45].

### Limitations

The main limitations have already been mentioned and justified in the preceding study [2], in particular the unsteadiness of the data over time: coverage and consistency of the data on the determinants. The coverage reaches 98% of all hospitalisations in public hospitals of the SNHS [46], while the consistency of the variables was stable over time [2]. Another limitation that has been mentioned concerns the activities that are recorded in PHE but not studied because they are not included in the dataset. These include emergency departments, outpatient non-surgical care-day services, or outpatient specialist consultations. However, as the healthcare professionals who support these services are included as factor of production in the personnel figure, we can consider them to be partially represented.

Another possible limitation of this study could come from the quarterisation based on the average APR-DRG weight per hospital, which might not assign some hospitals to the real group they belong to, especially those at the edges of the quartiles. Nevertheless, the inclusion of acute-care hospitals to one group or another was subsequently validated by the expert partners of the AtlasVPM group, who belong to the different Spanish regions and have in-depth knowledge of the reality of the hospitals in their localities.

Finally, with regard to the methodology used, information criteria were used to select between the different estimated models, and a set of validation tests were carried out to assess the compliance with the basic underlying hypothesis, which guarantees the validity of the inferences. In addition, the usual measures of goodness-of-fit were found to be above 99% (Fig 2)

Although not a limitation, it should be mentioned that the time series used for this study was truncated in 2015 due to the inconsistency of coded registries in 2016 and 2017 due to the change from ICD-9MC to ICD-10 coding. Although it does not include more current data, the time series analysed is sufficiently long enough and covers different events (the process of devolution of healthcare competences to regions and the financial crisis) for its validity to be considered.

### Implications

For 76% of hospitals in the first three quartiles of the complexity distribution (Groups 1 to 3), PHE remained more or less inelastic to quasi-prices depending on the hospital group, which means that a variation of one percentage point in any of the determinants capturing factors of production would lead to a smaller percentage effect on PHE, as shown in previous studies [24, 25]. However, the whole set of hospitals showed an elasticity greater than or equal to one with respect to the factors that capture the utilisation of hospital services (quantities), medical and surgical, which means that variations in the volume of activity generate proportional or more than proportional variations in PHE. The main finding compared to previous studies is the unitary elasticity of PHE with respect to the number of functioning beds found in the group of hospitals dealing with the most complex episodes of care (Group 4), which consequently makes PHE elastic to quasi-prices as a whole for 24% of the SNHS hospital network, representing over 50% of the total expenditure.

In Spain, Group 1 acute-care hospitals are mainly county hospitals in less densely populated areas. These hospitals appear to be more responsive to the needs of the population (demand). In addition to their specialisation as centres providing specific services (day care, minor general surgery, and obstetrics) and dealing with uncomplicated acute episodes, many of them have been transformed into medical (non-surgical) hospitals dealing mainly with chronic conditions or fragile patients, mostly with an older average age. Hospitals in Groups 2 and 3, which are now increasingly focused on outpatient case-day surgeries, obtained higher coefficients for this utilisation factor, such that its variations have a proportionately greater impact on PHE than in the other two groups of hospitals. On the other hand, hospitals in Group 4, the so-called tertiary hospitals, located in the main cities of the regions, are the "flagships" which, due to their size and the diversity and complexity of the pathologies they treat, seem to weigh heavily on public healthcare spending. The PHE of these hospitals, despite showing an elasticity above one to quantities (surgical and medical activity), turned out to be more sensitive to factors related to quasi-prices (factors of productions). This finding suggests the need for new management measures to optimise productivity.

Translating the marginal effects to monetary units would mean that for average monthly expenditure in 2015, a 1% variation in any of the utilisation factors would imply an average variation in PHE of € 2.7 million in Group 1, € 4 million in Group 2, € 4.7 million in Group 3 and € 13.9 million in Group 4 (specified by item in Table 2). In relative percentage terms, this variation would be higher in Groups 4 and 1. Meanwhile, a 1% variation in the statistically significant factors of production would imply a variation in PHE of € 4 million in Group 4, € 0.8 million in Group 3, and none in Groups 2 and 1.

**Table 2:** Impact on public hospital expenditure, by subgroup of hospitals, according to 1% variation in one of the specified determinants.

	Group1	Group2	Group3	Group4
surgical admissions	966,924 €	1,704,237 €	2,006,455 €	7,587,191 €
medical hospitalisations	1,419,686 €	1,550,432 €	1,846,132 €	5,021,802 €
outpatient case-day surgeries	330,009 €	717,408 €	810,578 €	1,285,426 €
Length of stay	**---**	**---**	**---**	1,797,263 €
Operative beds per-hospital	**---**	**---**	761,112 €	13,668,474 €
Professionals in context to beds	**---**	**---**	747,351 €	4,715,800 €

When jointly considering variations in the factors of production (quasi-prices) in hospitals dealing with de most complex episodes (Group 4), PHE proved to be the most sensitive to changes; specifying by item, a 1% variation in the average length of stay would imply a variation in PHE of € 0.4 million, a 1% variation in the average number of functioning beds per hospital would imply a variation in PHE of 2.8 million, and a 1% variation in the ratio of staff to beds would imply a variation in PHE of € 0.95 million (Table 2).

## Conclusions

The null hypothesis was not fulfilled: belonging to one or another cluster of hospitals mean that the determinant factors of expenditure affected them differently as well as with different intensity. While hospitals belonging to Groups 1 to 3 –accounting for 47,1% of total public hospital expenditure in 2015 –remained inelastic to quasi-prices factors to a greater or lesser extent, variations in the use of those factors of productions showed to be the main driver of PHE in hospitals belonging to Group 4, mainly due to the strong effect of the factor *number of operative beds per hospital*.

Meanwhile, factor quantities (utilisation factors) were the main drivers of the PHE interannual growth rate only in Groups 1 to 3, but not so in Group 4. However, in terms of intensity, the effect of the utilisation factors turned out to be stronger in the extreme groups (Groups 1 and 4), with elasticities above one compared to Groups 2 and 3 whose elasticities were equal one.

The findings showed how the Group 4 is the most sensitive to all the variables assessed. This highlights the need for promoting mechanisms to optimise their hospital activity given the existing resources in order to improve productivity and minimise its impact on hospital expenditure. Hospital performance improvement mechanisms, such as avoiding the practice of interventions in non-eligible patients, avoiding interventions with a more cost-effective alternative, or avoiding essentially ineffective interventions would have a more visible impact in hospitals belonging to Groups 1 to 3 but a more complex reorganisation processes would be needed in order to have an impact on PHE in Group 4 hospitals.

This suggests that the system focuses on these hospitals in order to optimally modulate PHE not only according to the needs of the population to be covered but also according to the current situation (giving them room for manoeuvre in the event of unforeseen events). At the same time it is important to complement it with a network of smaller hospitals (Group 1), close to their reference population, focused on managing and responding to chronicity and stabilisation, in case of acute events prior to transfer to tertiary hospitals (Group 4), which are larger but properly dimensioned and specialised in resolving acute and complex health issues.

## Supporting information

S1 Appendix Descriptive statistics and figures. (DOCX)

## References

[1] Gasto Sanitario. Informe Anual del Sistema Nacional de Salud 2017. Madrid. Ministry of Health, Consumption and Social Welfare. 2019. https://www.sanidad.gob.es/estadEstudios/estadisticas/sisInfSanSNS/tablasEstadisticas/InfAnualSNS2017/8_CAP_17.pdf [accessed 22nd May 2023].

[2] Bernal-Delgado E, Comendeiro-Maaløe M, Ridao-López M, Sansó-Roselló A. Factors Underlying the Growth of Hospital Expenditure in Spain in a Period of Unexpected Economic Shocks: A Dynamic Analysis on Administrative Data. Health Policy. 2020 Apr;124(4):389–396. doi: 10.1016/j.healthpol.2020.02.001 32063380

[3] General Health Act 14/1986 of 25 April (in Spanish: Ley 14/1986, de 25 de abril, General de Sanidad). https://www.boe.es/eli/es/l/1986/04/25/14/con (accessed 18 September 2019).

[4] Bernal-Delgado E, Garcia-Armesto S, Oliva J, Sánchez Martinez FI, Repullo JR, Pena-Longobardo LM, et al. Health Systems in Transition. 2018 May;20(2):1–179. http://www.euro.who.int/__data/assets/pdf_file/0008/378620/hit-spain-eng.pdf?ua=1 (accessed 24 June 2019). 30277216

[5] García-Armesto S, Begoña Abadía-Taira M, Durán A, Hernández-Quevedo C, Bernal-Delgado E. Spain: Health system review. Health Syst Transit. 2010;12(4):1–295, xix–xx 21224176

[6] Decentralisation Cantarero D. and health care expenditure: the Spanish case. Applied Economic Letters. 2005;12(15):963–966.

[7] Costa-Font J, Pons-Novell J. Public health expenditure and spatial interactions in a decentralized national health system. Health Economics. 2007; 16(3): 291–306. 16981194 10.1002/hec.1154

[8] Lopez-Casasnovas G, Costa-Font J, Planas I. Diversity and regional inequalities in the Spanish system of health care services. Health Economics. 2005; 14(S1): S221–235. 16161193 10.1002/hec.1038

[9] Cantarero Prieto D, Lago-Peñas S. Decomposing the determinants of health care expenditure: the case of Spain. European Journal of Health Economics. 2012;13(1): 19–27. 10.1007/s10198-010-0276-9 20853126

[10] Barnum H, Kutzin J. 1993 Public hospitals in developing countries: resource use, cost, financing. The International Bank. The Johns Hopkins University Press. USA. Available in: http://documentos.bancomundial.org/curated/es/919871468740383421/pdf/multi0page.pdf

[11] Disease control priorities in developing countries, 2nd edition. 2006. The International Bank for Reconstruction and Development / The World Bank; New York: Oxford University Press. 21250309

[12] https://www.mscbs.gob.es/estadEstudios/estadisticas/docs/NormaGRD2008/CLASIFICACIONHOSPITALESCLUSTER.pdf

[13] European Collaboration for Healthcare Optimization (ECHO) www.echo-health.eu. Zaragoza (Spain): Instituto Aragonés de Ciencias de la Salud-Instituto Investigación Sanitaria Aragón; c2011. Bernal-Delgado E, Thygesen LC, Martínez-Lizaga N, Comendeiro-Maaløe M, on behalf of the ECHO consortium. Handbook on methodology: Measurement of the variation; 2014 Apr 27; http://www.echo-health.eu/handbook/measuring_variation.html

[14] European Collaboration for Healthcare Optimization (ECHO) www.echo-health.eu Zaragoza (Spain): Instituto Aragonés de Ciencias de la Salud—Instituto Investigación Sanitaria Aragón; c2011. Estupiñán F, Baixauli C, Bernal-Delgado E on behalf of the ECHO consortium. Handbook on methodology: ECHO information system quality report; 2014 Apr 27; http://www.echo-health.eu/handbook/infrastructure.html

[15] Kelley E, Hurst J. OECD Health Care Quality Indicators Project. Conceptual framework paper. DELSA/HEA/WD/HWP(2006)3. Disponible en: https://www.oecd.org/els/health-systems/36262363.pdf

[16] Nolte E, Bain C, McKee M. “Population health” in Performance Measurement for Health System Improvement: Experiences, Challenges and Prospects. European Observatory on Health Systems and Policies. Ed.: Smith PC, Mossialos E, Papanicolas I, Leatherman S. Disponible en: http://www.euro.who.int/en/about-us/partners/observatory/publications/studies/performance-measurement-for-health-system-improvement-experiences,-challenges-and-prospects.

[17] Atlas de Variaciones de la Práctica Médica—Atlas VPM: http://www.atlasvpm.org/grupo-atlas-vpm.

[18] Burke LG, Khullar D, Zheng J, Frakt AB, Orav EJ, Jha AK Comparison of Costs of Care for Medicare Patients Hospitalized in Teaching and Nonteaching Hospitals. JAMA Netw Open. 2019 Jun 5;2(6):e195229. doi: 10.1001/jamanetworkopen.2019.5229 31173121 PMC6563581

[19] Newhouse JP. Accounting for teaching hospitals’ higher costs and what to do about them. Health Aff (Millwood). 2003;22(6):126–129. doi: 10.1377/hlthaff.22.6.126 14649439

[20] Bjorvatn A. Private or public hospital ownership: Does it really matter?. Soc Sci Med. 2018; 196:166–174. doi: 10.1016/j.socscimed.2017.11.038 29190537

[21] Akintoye E, Briasoulis A, Egbe A, Orhurhu V, Ibrahim W, Kumar K, et al. Effect of Hospital Ownership on Outcomes of Heart Failure Hospitalization. Am J Cardiol. 2017 Sep 1;120(5):831–837. doi: 10.1016/j.amjcard.2017.06.009 28689752

[22] Comendeiro-Maaløe M, Ridao-López M, Gorgemans S, Bernal-Delgado E Public-private partnerships in the Spanish National Health System: The reversion of the Alzira model. Health Policy. 2019 Apr;123(4):408–411. doi: doi: 10.1016/j.healthpol.2019.01.012 30739817

[23] Comendeiro-Maaløe M, Ridao-López M, Gorgemans S, Bernal-Delgado E. A comparative performance analysis of a renowned public private partnership for health care provision in Spain between 2003 and 2015. Health Policy. 2019 Apr;123(4):412–418. doi: 10.1016/j.healthpol.2018.11.009 30554791

[24] Gorgemans S, Comendeiro-Maaløe M, Ridao-López M, Enrique Bernal-Delgado E. Quality and Technical Efficiency Do Not Evolve Hand in Hand in Spanish Hospitals: Observational Study With Administrative Data. PLoS One 2018 Aug 2;13(8):e0201466. doi: 10.1371/journal.pone.0201466 30071062 PMC6072019

[25] Ridao-López M, Comendeiro-Maaløe M, Martínez-Lizaga N, Bernal-Delgado E. Evolution of public hospitals expenditure by healthcare area in the Spanish National Health System: the determinants to pay attention to. BMC Health Serv Res. 2018 Sep 10;18(1):696. doi: 10.1186/s12913-018-3445-7 30200956 PMC6131833

[26] Tonelli M, Wiebe N, Manns BJ, et al. Comparison of the Complexity of Patients Seen by Different Medical Subspecialists in a Universal Health Care System. JAMA Netw Open. 2018;1(7):e184852. doi: 10.1001/jamanetworkopen.2018.4852 30646392 PMC6324421

[27] Atlas de variaciones en la práctica médica. https://www.atlasvpm.org/en (accessed 20 January 2022)

[28] Estadística de centros sanitarios de atención especializada. Ministerio de Sanidad, Consumo y Bienestar Social. https://www.sanidad.gob.es/en/estadisticas/microdatos.do (accessed 20 July 2022).

[29] Hal R. Varian. Intermediate Microeconomics: A modern approach, 8th ed. 2010. Edi-tor: Jack Repcheck. https://faculty.ksu.edu.sa/sites/default/files/microeco-_varian.pdf (accessed 20 January 2022).

[30] Angulo-Pueyo E, Ridao-López M, Martínez-Lizaga N, García-Armesto S, PeiróS, Bernal-Delgado E. Factors associated with hospitalisations in chronic condi-tions deemed avoidable: ecological study in the Spanish healthcare system. BMJOpen 2017;7(2): e011844, https://bmjopen.bmj.com/content/9/4/e011844corr1. 10.1136/bmjopen-2016-011844 PMC5337668 28237952

[31] Geissler A, Scheller-Kreinsen D, Quentin W. Do diagnosis-related groupsapproximately explain variations in costs and lengths of stay of hip replace-ment? A comparative assessment of DRG systems across 10 Europeancountries. Health Economics 2012;21(Suppl. 52):103–15. Available at: doi: 10.1002/hec.2848 (accessed 20 January 2022). 22815116

[32] Street A, Kobel C, Renaud T, Thuilliez J. How well do Diagnosis RelatedGroups explain variation in costs and length of stay among patients andacross hospitals: methods for analysing routine patient data. Health Economics 2012;21(Suppl. 2):6–18. 22815108 10.1002/hec.2837

[33] Sin CY, White H. Information criteria for selecting possibly misspecified para-metric models. Journal of Econometrics 1996;71(1):207–25.

[34] Jarque CM, Bera AK. A test for normality of observations and regression residuals. International Statistical Review 1987;55(2):163–72.

[35] Fisher ES, Wennberg DE, Stukel TA, Gottlieb DJ, Lucas FL, Pinder EL. The impli-cations of regional variations in Medicare spending: part 1: the content, qualityand accessibility of care. Annals of Internal Medicine 2003;138:273–87. 12585825 10.7326/0003-4819-138-4-200302180-00006

[36] Fisher ES, Wennberg DE, Stukel TA, Gottlieb DJ, Lucas FL, Pinder EL. The impli-cations of regional variations in Medicare spending: part 2: health outcomes and satisfaction with care. Annals of Internal Medicine 2003;138:288–98 12585826 10.7326/0003-4819-138-4-200302180-00007

[37] Gottlieb DJ, Zhou W, Song Y, Andrews KG, Skinner JS, Sutherland JM. Pricesdon’t drive regional Medicare spending variations. Health Affairs (Millwood) 2010;29:537–43 doi: 10.1377/hlthaff.2009.0609 20110290 PMC2919810

[38] Curto V, Einav L, Finkelstein A, Levin JD, Bhattacharya J. Healthcare spending and utilization in public and private medicare. Cambridge: NBER; 2017, 10.3386/w23090 (January 2017) NBER Working Paper No. 23090

[39] Chandra A, Staiger DO. Productivity spillovers in health care: evidence from thetreatment of heart attacks. Journal of Political Economy 2007;115(1):103–40.[32] Skinner J. Causes and consequences of regional variations in health care. Hand-book of health economics, Vol 2. Amsterdam: Elsevier; 2007. p. 45–93.18418468

[40] Finkelstein A, Gentzkow M, Williams H. Sources of geographic variation inhealth care: evidence from patient migration. Quarterly Journal of Economics 2016;131(4):1681–726. doi: 10.1093/qje/qjw023 28111482 PMC5243120

[41] Newhouse JP, Garber AM, Graham RP, McCoy MA, Mancher M, Kibria A. Variation in health care spending: target decision mak-ing, not Geography. Institute of Medicine. Washington, D.C: The National Academies Press; 2013 https://www.nap.edu/catalog/18393/variation-in-health-care-spending-target-decision-making-not-geography 24851301

[42] Papanicolas I, Woskie LR, Jha AK. Health care spending in the United States and other high-income countries. JAMA 2018;319(10):1024–39, doi: 10.1001/jama.2018.1150 29536101

[43] Silber JH, Rosenbaum PR, Niknam BA, Ross RN, Reiter JG, Hill AS et al. Comparing Outcomes and Costs of Surgical Patients Treated at Major Teaching and Nonteaching Hospitals: A National Matched Analysis. Ann Surg.2020;271(3):412–421. doi: 10.1097/SLA.0000000000003602 31639108

[44] Pradarelli JC, Scally CP, Hari Nathan H, Thumma JR, Dimick JB. Hospital Teaching Status and Medicare Expenditures for Complex Surgery. Ann Surg. 2017 Mar; 265:502–513. doi: 10.1097/SLA.0000000000001706 28169925

[45] Abusaada K, Alsaleh L, Herrera V, Du Y, Baig H, Everett G. Comparison of Hospital Outcomes and Resource Use in Acute COPD Exacerbation Patients Managed by Teaching Versus Nonteaching Services in a Community Hospital. J Eval Clin Pract. 2017 Jun;23(3):625–630. doi: 10.1111/jep.12688 28054447

[46] Tebé C, Abilleira S, Ridao M, Espallargues M, Salas T, Bernal-Delgado E y Atlas. Atlas de Variaciones en el manejo de la Enfermedad Cerebrovascular Isquémica 2013;5:389–424

