# Supplementary material for: Delving into public-expenditure elasticity: Evidence from a National Health Service acute-care hospital network
Source: PLoS One. 2024 Mar 4;19(3):e0291991. doi: 10.1371/journal.pone.0291991 (PMC10911587; doi:10.1371/journal.pone.0291991)
Supplement: S1 Appendix — (DOCX) [file pone.0291991.s001.docx]

**S1 Analytical supporting appendix – Descriptive statistics and figures**

S1 Table 1. Descriptive statistics

|  |  | **mean** | **Std. Dev** | **min** | **max** | **p5** | **p50** | **p95** | **CV** |
| --- | --- | --- | --- | --- | --- | --- | --- | --- | --- |
| **Deflated, severity-adjusted, monthly public hospital expenditure (Million)** | Cgr1 | 216.4 € | 44.7 € | 120.4 € | 306.3 € | 141.4 € | 223.5 € | 278.4 € | 0.21 |
|  | Cgr2 | 343.9 € | 60.9 € | 198.8 € | 457.7 € | 239.6 € | 356.2 € | 429.2 € | 0.18 |
|  | Cgr3 | 392.3 € | 77.8 € | 205.7 € | 534.2 € | 261.7 € | 405.1 € | 499.5 € | 0.20 |
|  | Cgr4 | 1,160.5 € | 168.8 € | 683.9 € | 1,502.4 € | 874.3 € | 1,185.2 € | 1,410.3 € | 0.15 |
| **Percentage of men** | Cgr1 | 0.466 | 0.005 | 0.450 | 0.477 | 0.455 | 0.466 | 0.474 | 0.01 |
|  | Cgr2 | 0.468 | 0.007 | 0.450 | 0.483 | 0.458 | 0.466 | 0.479 | 0.02 |
|  | Cgr3 | 0.467 | 0.006 | 0.453 | 0.481 | 0.457 | 0.467 | 0.477 | 0.01 |
|  | Cgr4 | 0.472 | 0.005 | 0.459 | 0.483 | 0.464 | 0.472 | 0.480 | 0.01 |
| **Ageing index (people ≥ 65 to people ≤ 15)** | Cgr1 | 4.321 | 0.676 | 3.177 | 5.772 | 3.343 | 4.372 | 5.314 | 0.16 |
|  | Cgr2 | 4.729 | 0.774 | 3.333 | 6.514 | 3.595 | 4.686 | 6.076 | 0.16 |
|  | Cgr3 | 5.761 | 0.819 | 4.290 | 7.984 | 4.498 | 5.780 | 7.223 | 0.14 |
|  | Cgr4 | 4.221 | 0.624 | 3.278 | 5.838 | 3.368 | 4.191 | 5.312 | 0.15 |
| **Over-ageing index 2 (people ≥ 75 to people ≥ 65)** | Cgr1 | 0.642 | 0.027 | 0.579 | 0.691 | 0.589 | 0.647 | 0.684 | 0.04 |
|  | Cgr2 | 0.631 | 0.028 | 0.561 | 0.681 | 0.577 | 0.637 | 0.673 | 0.04 |
|  | Cgr3 | 0.623 | 0.027 | 0.562 | 0.671 | 0.573 | 0.629 | 0.668 | 0.04 |
|  | Cgr4 | 0.600 | 0.030 | 0.531 | 0.649 | 0.542 | 0.609 | 0.645 | 0.05 |
| **Morbidity (average)** | Cgr1 | 1622.4 | 96.3 | 1319.0 | 1883.0 | 1436.0 | 1618.5 | 1781.0 | 0.06 |
|  | Cgr2 | 2649.1 | 163.6 | 2307.0 | 3035.0 | 2366.0 | 2646.5 | 2926.0 | 0.06 |
|  | Cgr3 | 2893.2 | 232.6 | 2325.0 | 3465.0 | 2505.0 | 2915.5 | 3244.0 | 0.08 |
|  | Cgr4 | 7126.1 | 458.5 | 5905.0 | 8269.0 | 6335.0 | 7124.0 | 7820.0 | 0.06 |
| **Average monthly amount of hospitals** | Cgr1 | 63.8 | 5.0 | 56.0 | 69.0 | 56.0 | 66.0 | 69.0 | 0.08 |
|  | Cgr2 | 66.1 | 3.4 | 61.0 | 69.0 | 61.0 | 68.0 | 69.0 | 0.05 |
|  | Cgr3 | 59.6 | 6.8 | 49.0 | 69.0 | 49.0 | 61.0 | 69.0 | 0.11 |
|  | Cgr4 | 63.8 | 1.8 | 60.0 | 66.0 | 61.0 | 64.0 | 66.0 | 0.03 |
| **Surgical hospitalisations** | Cgr1 | 12170.3 | 1513.3 | 8349.0 | 15272.0 | 9488.0 | 12392.0 | 14352.0 | 0.12 |
|  | Cgr2 | 18987.4 | 2356.9 | 13500.0 | 23382.0 | 14239.0 | 19406.0 | 22191.0 | 0.12 |
|  | Cgr3 | 20340.5 | 2831.2 | 12462.0 | 24900.0 | 14642.0 | 20863.5 | 23968.0 | 0.14 |
|  | Cgr4 | 56683.2 | 6922.1 | 36341.0 | 68874.0 | 42100.0 | 57825.5 | 65572.0 | 0.12 |
| **Medical hospitalisations** | Cgr1 | 25295.6 | 1796.9 | 21533.0 | 30908.0 | 22562.0 | 25135.5 | 28708.0 | 0.07 |
|  | Cgr2 | 37105.2 | 2359.7 | 32635.0 | 43658.0 | 33453.0 | 36980.5 | 41775.0 | 0.06 |
|  | Cgr3 | 38474.2 | 2965.7 | 32195.0 | 47287.0 | 33534.0 | 38418.5 | 43442.0 | 0.08 |
|  | Cgr4 | 91121.2 | 5561.1 | 77177.0 | 105933.0 | 82045.0 | 91604.0 | 100049.0 | 0.06 |
| **Outpatient day care surgery** | Cgr1 | 11954.2 | 4980.3 | 2202.0 | 22309.0 | 4961.0 | 11593.0 | 20432.0 | 0.42 |
|  | Cgr2 | 18594.0 | 7628.4 | 3413.0 | 35158.0 | 7099.0 | 17593.5 | 32001.0 | 0.41 |
|  | Cgr3 | 16144.3 | 5770.2 | 3779.0 | 30039.0 | 7857.0 | 15738.0 | 26144.0 | 0.36 |
|  | Cgr4 | 35758.7 | 10913.4 | 6867.0 | 55770.0 | 16166.0 | 36844.5 | 53807.0 | 0.31 |
| **Average length of stay in medical hospitalisations** | Cgr1 | 5.96 | 0.24 | 5.48 | 6.67 | 5.58 | 5.94 | 6.42 | 0.04 |
|  | Cgr2 | 6.62 | 0.22 | 5.99 | 7.24 | 6.30 | 6.63 | 7.01 | 0.03 |
|  | Cgr3 | 6.65 | 0.24 | 6.04 | 7.21 | 6.29 | 6.65 | 7.05 | 0.04 |
|  | Cgr4 | 7.30 | 0.36 | 6.31 | 8.13 | 6.76 | 7.27 | 7.89 | 0.05 |
| **Average length of stay in surgical hospitalisations** | Cgr1 | 5.78 | 0.38 | 4.88 | 6.96 | 5.21 | 5.76 | 6.48 | 0.07 |
|  | Cgr2 | 6.24 | 0.43 | 4.94 | 7.26 | 5.58 | 6.25 | 6.95 | 0.07 |
|  | Cgr3 | 6.59 | 0.47 | 5.31 | 8.12 | 5.93 | 6.59 | 7.30 | 0.07 |
|  | Cgr4 | 8.39 | 0.70 | 6.17 | 10.39 | 7.31 | 8.43 | 9.54 | 0.08 |
| **Average monthly amount of functioning beds** | Cgr1 | 238.40 | 4.65 | 229.44 | 247.51 | 231.39 | 239.25 | 245.58 | 0.02 |
|  | Cgr2 | 332.16 | 8.61 | 314.87 | 347.82 | 317.98 | 335.20 | 344.23 | 0.03 |
|  | Cgr3 | 461.82 | 20.21 | 425.93 | 504.99 | 431.41 | 457.37 | 489.54 | 0.04 |
|  | Cgr4 | 883.66 | 58.69 | 799.17 | 961.63 | 802.89 | 893.11 | 955.70 | 0.07 |
| **Ratio medical staff to functioning beds Average monthly fully contracted doctors** | Cgr1 | 0.70 | 0.06 | 0.57 | 0.78 | 0.57 | 0.72 | 0.78 | 0.09 |
|  |  | 166 | 15 | 133 | 187 | 134 | 172 | 184 | 0.09 |
|  | Cgr2 | 0.69 | 0.07 | 0.57 | 0.78 | 0.57 | 0.73 | 0.78 | 0.11 |
|  |  | 231 | 25 | 181 | 263 | 182 | 244 | 258 | 0.11 |
|  | Cgr3 | 0.67 | 0.07 | 0.56 | 0.76 | 0.56 | 0.68 | 0.76 | 0.10 |
|  |  | 308 | 21 | 269 | 351 | 273 | 310 | 340 | 0.07 |
|  | Cgr4 | 0.71 | 0.07 | 0.59 | 0.80 | 0.59 | 0.75 | 0.79 | 0.09 |
|  |  | 627 | 32 | 555 | 680 | 568 | 631 | 675 | 0.05 |
| **Ratio nursing staff to functioning beds Average monthly fully contracted nursing staff** | Cgr1 | 1.12 | 0.07 | 0.98 | 1.23 | 0.99 | 1.15 | 1.23 | 0.06 |
|  |  | 268 | 18 | 228 | 296 | 230 | 272 | 293 | 0.07 |
|  | Cgr2 | 1.14 | 0.06 | 1.02 | 1.22 | 1.02 | 1.15 | 1.21 | 0.05 |
|  |  | 379 | 24 | 324 | 412 | 327 | 386 | 408 | 0.06 |
|  | Cgr3 | 1.11 | 0.05 | 1.02 | 1.17 | 1.02 | 1.13 | 1.17 | 0.05 |
|  |  | 513 | 15 | 484 | 554 | 490 | 516 | 535 | 0.03 |
|  | Cgr4 | 1.31 | 0.06 | 1.19 | 1.39 | 1.20 | 1.34 | 1.39 | 0.04 |
|  |  | 1159 | 58 | 1057 | 1249 | 1066 | 1155 | 1244 | 0.05 |
| **Ratio medical professionals in training (MIR) to total medical professionals Average monthly medical professionals in training** | Cgr1 | 0.14 | 0.03 | 0.10 | 0.19 | 0.11 | 0.13 | 0.19 | 0.20 |
|  |  | 24 | 7 | 14 | 34 | 14 | 22 | 33 | 0.27 |
|  | Cgr2 | 0.18 | 0.03 | 0.14 | 0.22 | 0.14 | 0.17 | 0.22 | 0.16 |
|  |  | 42 | 11 | 26 | 58 | 27 | 42 | 57 | 0.25 |
|  | Cgr3 | 0.23 | 0.03 | 0.18 | 0.27 | 0.18 | 0.22 | 0.27 | 0.12 |
|  |  | 71 | 12 | 48 | 87 | 49 | 67 | 86 | 0.17 |
|  | Cgr4 | 0.40 | 0.02 | 0.36 | 0.45 | 0.37 | 0.40 | 0.45 | 0.06 |
|  |  | 250 | 18 | 209 | 275 | 219 | 249 | 274 | 0.07 |

Std. Dev. stands for the Standard Deviation. CV stands for the coefficient of variation. Blue figures stand for averages.

S1 Fig 1. Evolution of monthly *need factors* by group of hospitals, Jan2003-Dec2015.


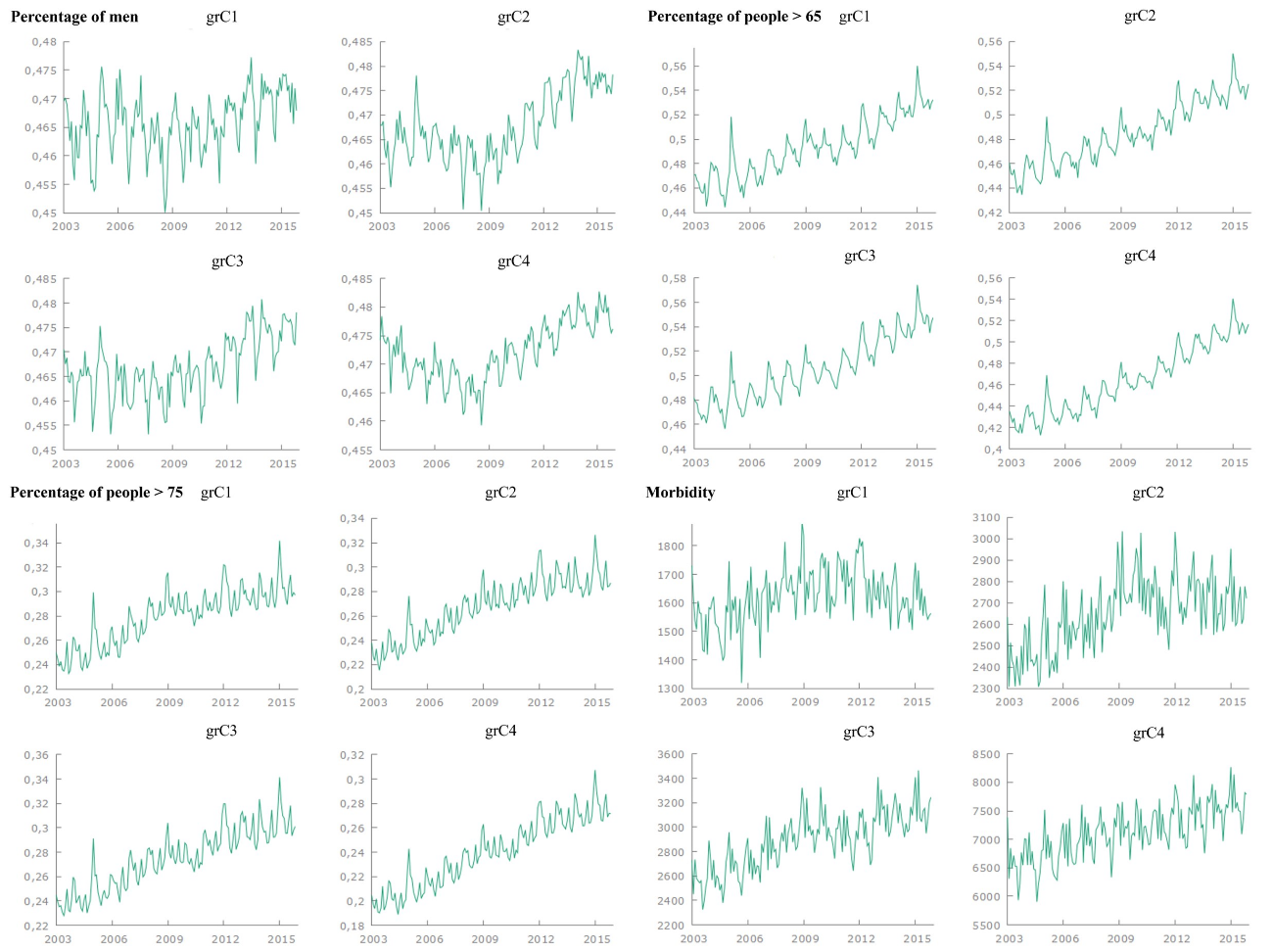


S1 Fig 2. Evolution of monthly *utilisation factors* by group of hospitals, Jan2003-Dec2015.


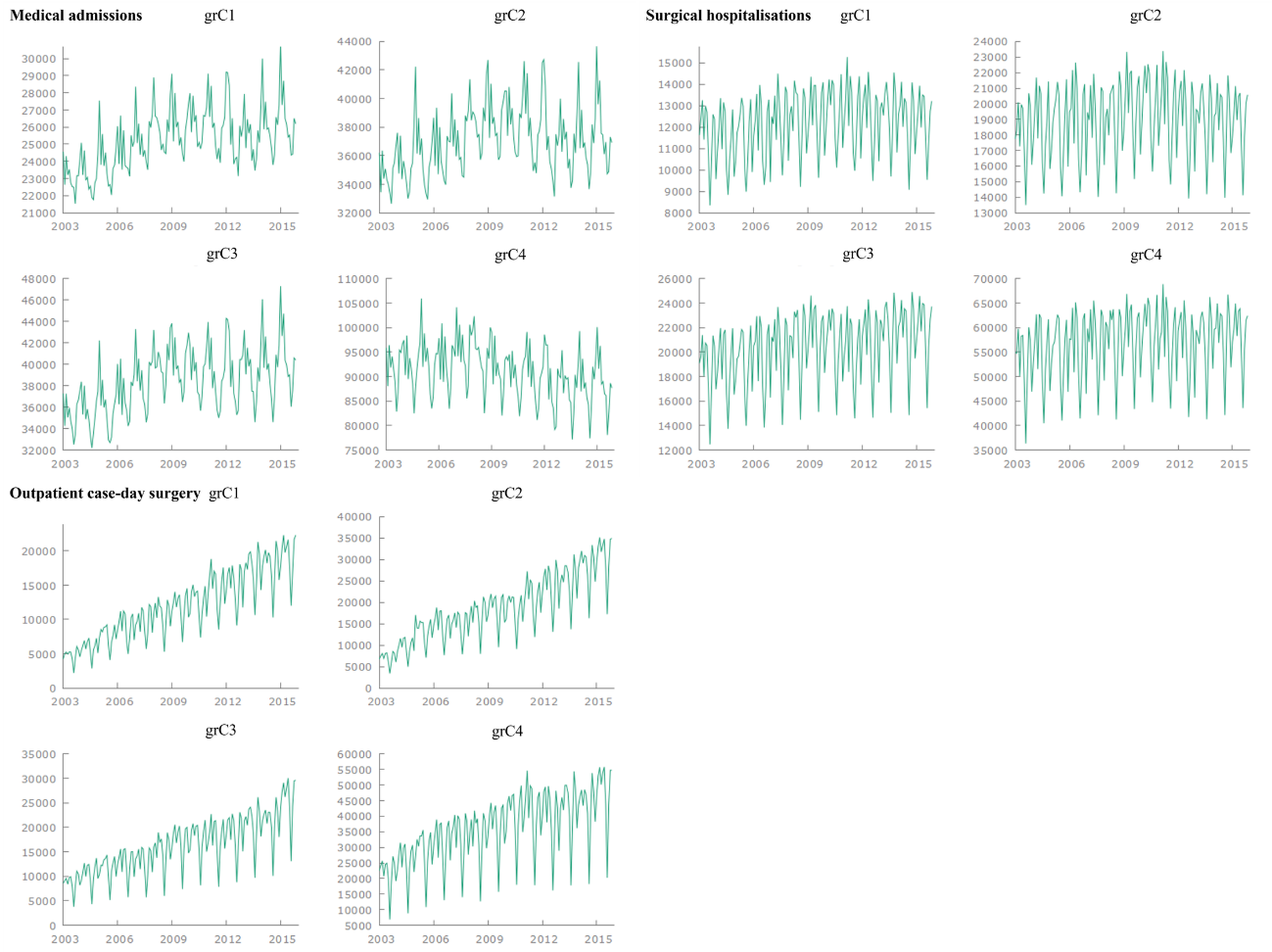


S1 Fig 3a. Evolution of factors of production (quasi-price factors) by group of hospitals, Jan2003-Dec2015.


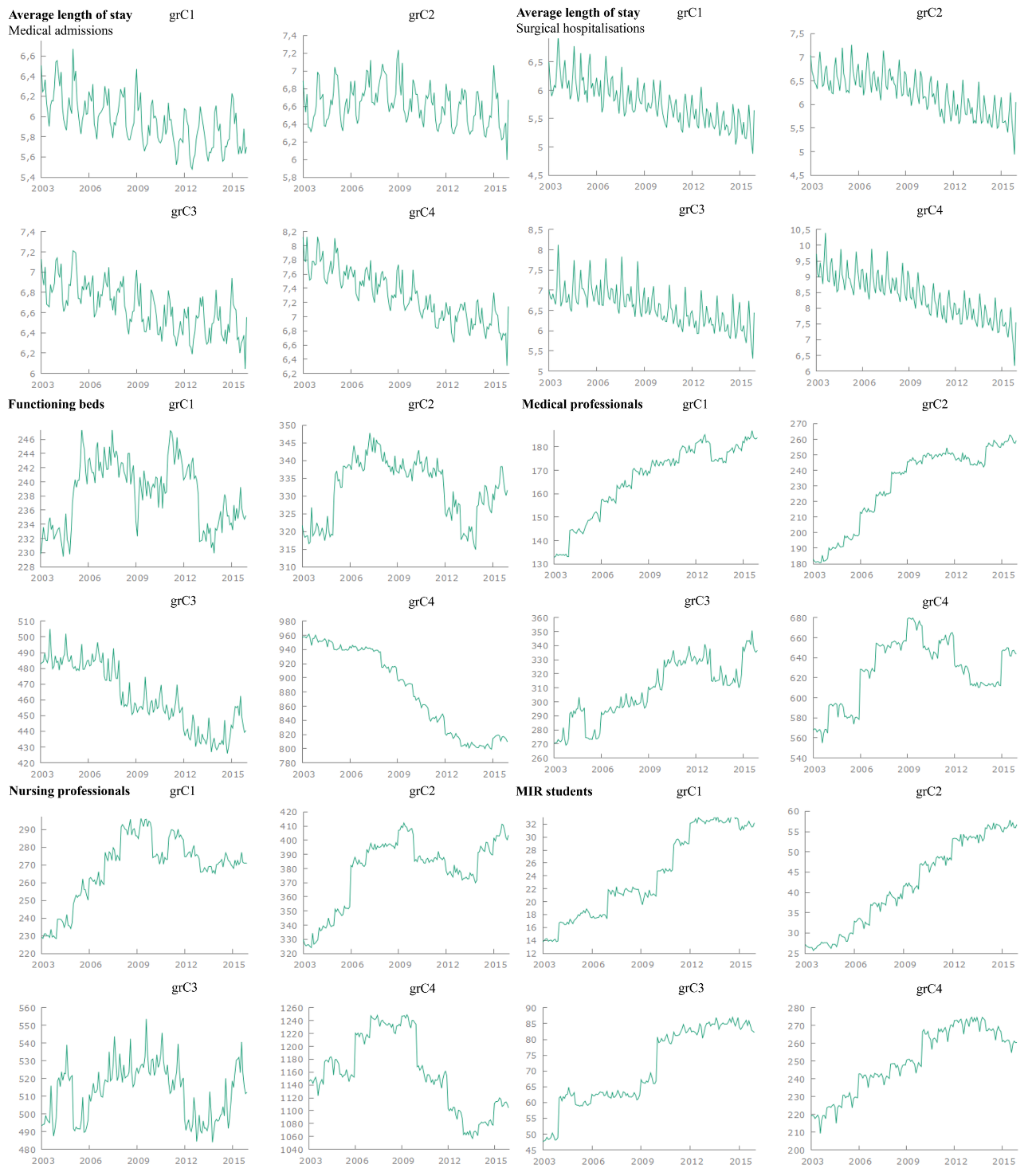


S1 Fig 3b. Evolution of factors of production (human resources) by group of hospitals in relation to beds, Jan2003-Dec2015.


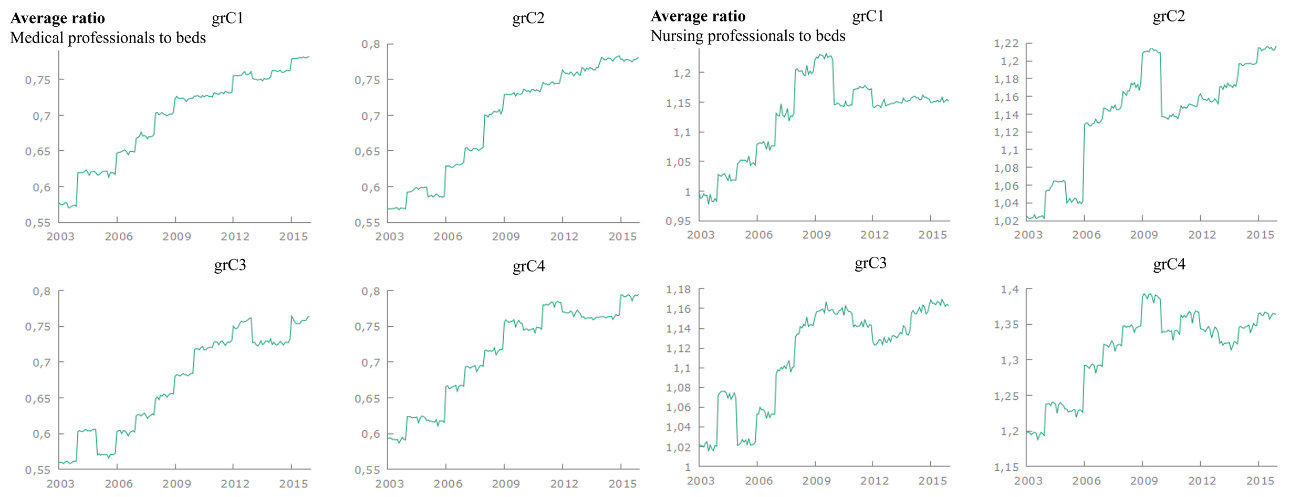


S1 Table 2. Deflated, severity adjusted, public hospital expenditure by subgroups of hospitals according to the severity of the episodes attended to.

Figures in parenthesis stand for p-values

| **SARIMAX regressions** | **Cgr1** | | **Cgr2** | | **Cgr3** | | **Cgr4** | |
| --- | --- | --- | --- | --- | --- | --- | --- | --- |
|  | SARIMAX (0,3)(0,1,1) | | SARIMAX(0-5,6,7)(1,1,0) | | SARIMAX (0,0)(1,1,0) | | SARIMAX (0,0)(1,1,0) | |
|  | Coefficient | Std. Dev | Coefficient | Std. Dev | Coefficient | Std. Dev | Coefficient | Std. Dev |
| Intercept | 0.0408 | 0.0055 | 0.0319 | 0.0039 | 0.0671 | 0.0064 | 0.0529 | 0.0045 |
|  |  | (0.0000) |  | (0.0000) |  | (0.0000) |  | (0.0000) |
| Percentage of men | 0.1693 | 0.1841 | -0.2660 | 0.1996 | 0.3107 | 0.1783 | -0.4209 | 0.2697 |
|  |  | (0.3577) |  | (0.1827) |  | (0.0814) |  | (0.1186) |
| Percentage over 65 | 1.3205 | 0.2254 | -0.3945 | 0.2377 | 0.0058 | 0.2272 | 0.2658 | 0.2685 |
|  |  | (0.0000) |  | (0.0970) |  | (0.9796) |  | (0.3222) |
| Percentage over 75 | -1.3340 | 0.2276 | 0.5157 | 0.2787 | 0.4628 | 0.2089 | 0.3234 | 0.2885 |
|  |  | (0.0000) |  | (0.0643) |  | (0.0267) |  | (0.2623) |
| Ln (morbidity) | -0.0318 | 0.0174 | 0.0050 | 0.0179 | 0.0102 | 0.0172 | -0.0572 | 0.0252 |
|  |  | (0.0668) |  | (0.7803) |  | (0.5545) |  | (0.0235) |
| Ln (surgical hospitalisations) | 0.3784 | 0.0235 | 0.4346 | 0.0209 | 0.4239 | 0.0178 | 0.6000 | 0.0311 |
|  |  | (0.0000) |  | (0.0000) |  | (0.0000) |  | (0.0000) |
| Ln (medical hospitalisations) | 0.5556 | 0.0300 | 0.3953 | 0.0298 | 0.3900 | 0.0242 | 0.3971 | 0.0340 |
|  |  | (0.0000) |  | (0.0000) |  | (0.0000) |  | (0.0000) |
| Ln (outpatient case-day surgery) | 0.1292 | 0.0127 | 0.1829 | 0.0104 | 0.1712 | 0.0104 | 0.1017 | 0.0147 |
|  |  | (0.0000) |  | (0.0000) |  | (0.0000) |  | (0.0000) |
| Ln (average length of stay in surgical hospitalisations) | 0.0543 | 0.0360 | 0.0487 | 0.0293 | 0.1144 | 0.0255 | 0.1152 | 0.0355 |
|  |  | (0.1314) |  | (0.0963) |  | (0.0000) |  | (0.0012) |
| Ln (average length of stay in medical hospitalisations) | 0.0301 | 0.0449 | 0.0051 | 0.0328 | -0.0904 | 0.0358 | 0.0270 | 0.0462 |
|  |  | (0.5028) |  | (0.8127) |  | (0.0116) |  | (0.5591) |
| Ln (number of hospitals in the cluster) | 0.2727 | 0.0890 | 0.1344 | 0.1349 | 0.0910 | 0.0322 | 0.1592 | 0.0758 |
|  |  | (0.0022) |  | (0.3188) |  | (0.0047) |  | (0.0356) |
| Ln (functioning beds) | -0.0012 | 0.0620 | 0.2007 | 0.0469 | 0.2518 | 0.0805 | 1.2401 | 0.0964 |
|  |  | (0.9850) |  | (0.0000) |  | (0.0018) |  | (0.0000) |
| Ln (medical professionals) | 0.3553 | 0.0550 | -1.6772 | 0.1902 | -0.1350 | 0.0271 | 0.4629 | 0.2327 |
|  |  | (0.0000) |  | (0.0000) |  | (0.0000) |  | (0.0000) |
| Ln (nursing staff) | -0.2670 | 0.0502 | 1.8079 | 0.1635 | 0.0700 | 0.0398 | -0.7255 | 0.1308 |
|  |  | (0.0000) |  | (0.0000) |  | (0.0790) |  | (0.0000) |
| Ln (Medical professionals in training) | -0.0659 | 0.0300 | -0.2392 | 0.0230 | -0.0289 | 0.0250 | -0.6046 | 0.0640 |
|  |  | (0.0282) |  | (0.0000) |  | (0.2474) |  | (0.0000) |
